# Influence of two pediatric multivitamin forms on surface characteristics and color stability of single-shade composite, nanofilled composite, and glass ionomer cement: an in vitro study

**DOI:** 10.1186/s12903-026-08673-0

**Published:** 2026-06-06

**Authors:** Mai M. Elbatanony, Amal M. El Shahawi, Dalia Y. Zaki

**Affiliations:** Restorative and Dental Materials Department, Oral and Dental Research Institute, National Research Centre, Cairo, Egypt

**Keywords:** Chewable multivitamins, Glass ionomer, Gummy multivitamins, Hardness, Omnichroma resin composite, Surface roughness

## Abstract

**Background:**

Gummy and chewable pediatric multivitamin formulations commonly contain citric acid and ascorbic acid, yielding pH values that may fall below the critical erosive threshold of 5.5. These organic acids can degrade restorative materials through proton-mediated surface dissolution and chelation of matrix cations. Despite widespread use, evidence on their long-term effect on contemporary restorative materials remains limited. This study aimed to assess the effect of gummy and chewable forms of pediatric multivitamin supplements on the surface hardness, roughness, and color stability of glass ionomer cement (Medifil), universal single-shade resin composite (Omnichroma), and a nanofilled composite resin (Filtek Z350 XT).

**Methods:**

Specimens (*n* = 135) of Omnichroma composite, Filtek Z350 XT composite, and Medifil glass ionomer cement were prepared and assigned to three subgroups (*n* = 15): (1) artificial saliva (control), (2) HerbaL Gummies dissolved in artificial saliva, and (3) Centrum chewable tablets dissolved in artificial saliva. After 6-month daily in vitro immersion cycles (69 s/day at 37 °C), surface hardness (VHN), roughness (Sa via AFM), and color change (ΔE) were evaluated. Data were analyzed using a two-way ANOVA with a 3 × 3 factorial design, followed by Tukey’s HSD post hoc test.

**Results:**

Two-way ANOVA revealed significant main effects of material and immersion medium, and significant material×medium interactions for all outcomes (all *p* ≤ 0.001). Both solutions significantly reduced hardness and increased roughness vs. control. Glass ionomer cement was the most susceptible material for surface hardness and roughness (lowest VHN and highest Sa across all conditions; *p* < 0.001 vs. both composites), while Filtek Z350 XT was the least affected. HerbaL Gummies produced greater hardness reduction than Centrum in glass ionomer and Filtek Z350 XT, while Omnichroma responded comparably to both. Omnichroma exhibited the greatest color change across all conditions (ΔE = 3.13), though all ΔE values remained clinically acceptable (ΔE < 3.3).

**Conclusion:**

Daily in vitro exposure to acidic pediatric multivitamin supplements adversely affected the surface hardness, roughness, and color stability of all tested restorative materials under simulated oral conditions, with material- and formulation-specific degradation patterns. HerbaL Gummies demonstrated greater erosive potential than Centrum chewable tablets for most material outcome combinations. Clinicians should account for supplement acidity when selecting restorative materials and should advise parents to administer supplements with meals and rinse with water immediately afterward.

## Introduction

Vitamins are essential for children’s health to ensure proper growth and maintain healthy tissue function. They are obtained from different external sources when adequate amounts cannot be provided through diet alone [1]. They are available in various forms, including syrups, pills, chewable tablets, gummies, and effervescent tablets [2].

Several types of vitamins are pH-dependent and are supplied as acidic formulations to ensure proper drug function, which may have an erosive effect on teeth [3]. Research has demonstrated the erosive effects of these supplements on enamel microhardness. Pasdar N. et al. [4], investigated the effect of iron and multivitamin drops with low pH values on the enamel microhardness of primary teeth. The results revealed that the studied supplements had erosive effects on teeth to varying degrees. Preventive measures were recommended after using these types of drops.

Other studies have shown that pediatric medications such as antitussives, analgesics, antibiotics, and antihistamines with high acidity have erosive effects on primary and permanent tooth enamel and may cause matrix degradation of resin composites. Several studies have reported a positive association between the frequency and duration of acidic pediatric supplement exposure and the degree of enamel erosion, with liquid formulations demonstrating greater erosive potential than solid forms due to their more prolonged contact with tooth surfaces. It should be noted, however, that the magnitude of the erosive effect varies across studies depending on the specific formulation examined, the pH and titratable acidity of the preparation, the duration and frequency of exposure, and whether primary or permanent dentition was investigated [3, 5–10].

While the erosive impact of acidic pediatric supplements on hard dental tissues is well established, the clinical significance of this phenomenon extends beyond enamel alone. Children who require esthetic dental restorations, including glass ionomer cements and resin composites, are frequently the same patients who routinely consume multivitamin preparations. The acidic environment generated by these supplements within the oral cavity therefore does not act exclusively on tooth structure; it simultaneously contacts any restorative material present. Unlike hydroxyapatite, which dissolves through a relatively uniform acid–base mechanism, tooth-colored restorative materials are susceptible to degradation through distinct and material-specific pathways: proton-mediated disruption of polyacrylate cross-links and glass-phase dissolution in glass ionomer cements, and acid-facilitated water sorption, monomer leaching, and filler–matrix interface breakdown in resin composites. These material-specific degradation pathways manifest clinically through distinct and measurable consequences, as elaborated below.

Surface characteristics including hardness, roughness, and color stability are key determinants of the clinical longevity and esthetic performance of tooth-colored restorative materials [11]. Acidic environments are well-established contributors to the degradation of both tooth structure and restorative materials, including glass ionomer cements and composite resins [12]. The pH values of multivitamin supplements that fall below the critical threshold of 5.5 may initiate softening and structural degradation of restorative materials [1]. This acidic profile is attributable to the presence of ascorbic acid, citric acid, and related compounds, which are incorporated into both gummy and chewable tablet formulations to enhance flavor, stability, and bioavailability of active ingredients [13].

With respect to surface hardness, proton-mediated disruption of the polyacrylate cross-links in glass ionomer cements and acid-facilitated water sorption, monomer leaching, and plasticization of the polymer network in resin composites can progressively reduce the mechanical integrity of the restoration surface, increasing susceptibility to wear and deformation under masticatory forces [14].

Regarding surface roughness, acid-induced filler particle displacement and matrix degradation produce surface irregularities with direct clinical consequences; a surface roughness threshold of 0.2 μm has been established as the clinically relevant limit above which bacterial plaque retention and biofilm formation are significantly promoted, increasing the risk of secondary caries and periodontal inflammation around restored teeth [15]. Color stability represents a further and mechanistically distinct concern; unlike hardness and roughness changes, color alteration is not a direct consequence of erosion per se but rather results from surface degradation-induced microporosity that facilitates the penetration and entrapment of chromogenic compounds within the restoration surface [8]. This is of clinical significance in pediatric dentistry, where esthetic outcomes are increasingly prioritized by both patients and parents, and where long-term color stability directly influences patient satisfaction and restoration longevity.

Since esthetics have become one of the primary demands in dental clinics, even for children, different types of restorative materials have been introduced. Glass ionomer cement materials, with their specific features of ion release, chemical bonding, and ease of manipulation, as well as composite restorative materials, are reliable choices for esthetic restorations in children. However, restoration discoloration may occur depending on the degree of polymerization, filler size and shape, and the consumption of foods, beverages, and medications [16–20].

In recent years, a universal single-shade resin composite (Omnichroma) was introduced to the dental market. It is characterized by uniformly sized nanospherical fillers and incorporates smart chromatic technology that enables it to match all VITA Classical shades using a single resin composite shade. This wide range of shade matching has been reported to reduce clinical steps, minimize shade-matching errors, and decrease the number of resin composite shades required [21–23].

The durability of esthetic properties and surface characteristics of restorative materials against the harsh intraoral environment, including temperature fluctuations and pH changes, is essential. These conditions may cause mechanical wear along with alterations in structural and surface characteristics [24].

Despite the documented erosive potential of acidic pediatric medications and vitamin supplements on hard dental tissues, the available evidence remains largely confined to enamel substrates or conventional restorative materials. Limited attention has been given to the behavior of glass ionomer cements and, more importantly, recently introduced universal single-shade resin composites that rely on structural color technology rather than pigment-based shade systems. To date, no studies have comprehensively evaluated the effect of commonly consumed pediatric multivitamin formulations, particularly gummy and chewable tablet forms, on the surface hardness, surface roughness, and color stability of these contemporary restorative materials under long-term simulated oral conditions. Therefore, there is a clear gap in the literature regarding the long-term surface and esthetic stability of modern restorative materials when exposed to commonly used pediatric multivitamin preparations.

The aim of this study was to assess the effect of two common forms of multivitamins for children on the surface hardness, surface roughness, and color change of three types of direct restorative materials: glass ionomer cements, a universal single-shade resin composite (Omnichroma), and nanofilled composite resin restorative materials. The null hypothesis was that the tested multivitamins would not affect the surface hardness or roughness, nor cause color change in the tested esthetic restorative materials.

## Materials and methods

### Restorative materials

Three types of direct restorative materials were tested in this study. Their manufacturers, compositions, shades, and lot numbers are listed in Table 1.

**Table 1:** Materials used in the study, including their manufacturers, compositions, shades, and lot numbers

Product	Manufacturer	Composition	Shade	Lot number
Medifil glass ionomer filling material (GI)	Promedica Dental Material GmbH (Neumünster, Germany)	Powder: 95% strontium fluoro alumino silicate (FAS) glass + 5% freeze-dried (anhydrous) polyacrylic acid. Liquid: 30 wt% aqueous polyacrylic acid solution.	A3	2,150,498
Omnichroma composite restorative material (OM)	Tokuyama Dental Corporation (Tokyo, Japan)	Filler: 79 wt% consisting of uniformly sized 260 nm spherical SiO₂-ZrO₂ supra-nano spherical fillers and pre-polymerized composite fillers. Matrix: UDMA, TEGDMA.	Universal	O42EZ1
Filtek Z350 XT nanofilled composite restorative material (CR)	3 M ESPE (St. Paul, MN, USA)	Fillers: 78.5 wt% non-agglomerated/non-aggregated 20 nm silica filler, 4–11 nm zirconia filler, and aggregated zirconia/silica cluster filler. Matrix: Bis-GMA, BisEMA, UDMA, TEGDMA.	Enamel A2	NE57549

### The Multivitamin types

Two different forms of multivitamins were used in this study. Their ingredients, manufacturers, and lot numbers are listed in Table 2.

**Table 2:** Multivitamin types used in the study including their ingredients, manufacturers, and lot numbers

Multivitamin type	Ingredients	Manufacturer	Lot number	pH
HerbaL Gummies for Kids (HG)	Vitamin A (Palmitate) 786 mcg RAE; Vitamin C (Ascorbic Acid) 20 mg; Vitamin D3 6.0 mcg; Vitamin E 7.4 mg; Vitamin B6 1.04 mg; Folic Acid 260 mcg; Vitamin B12 5.1 mcg; Biotin 60 mcg; Pantothenic Acid 5.2 mg; Iodine 42 mcg; Zinc 2.7 mg; Choline 40 mcg; Inositol 40 mcg. Other ingredients: Cane Sugar, Organic Tapioca Syrup, Purified Water, Pectin, Citric Acid, Sodium Citrate, Natural Flavors, Natural Colors, Organic Sunflower Oil.	HerbaL and Naturals Inc. (Richmond, BC, Canada)	80,066,504	4.7
Centrum Kids Chewable Multivitamin (CT)	Retinol acetate 450 mcg; Vitamin D3 2.5 mcg; Calcium pantothenate 4.5 mg; Thiamine nitrate 1 mg; Nicotinamide 15 mg; Riboflavine 1.2 mg; Pyridoxine HCl 2 mg; Cyanocobalamin 1.2 mcg; Biotin 50 mcg; Folic acid 100 mcg; Vitamin E 10 mg; Ascorbic acid 50 mg; Calcium 12 mg; Phosphorus 9.275 mg; Magnesium 5 mg; Iron 1.5 mg; Zinc 1 mg; Manganese 1 mg. Other ingredients: Sugar, Citric Acid, Stearic Acid.	Haleon (Warren, NJ, USA)	8699DN	5.1

### Specimen preparation

For surface hardness and surface roughness testing, disc-shaped Teflon molds (5 mm × 2 mm) were used. For color change testing, molds measuring 10 mm × 1 mm were used. The larger diameter (10 mm) for color change specimens was selected to ensure complete and unobstructed coverage of the spectrophotometer measurement aperture, preventing edge-related light scattering artifacts and measurement errors. The reduced thickness (1 mm) was adopted to standardize the optical path length through the specimen and minimize the influence of material translucency on recorded L*, a*, and b* values, consistent with ISO 7491:2000 guidelines and widely adopted protocols in dental color stability research [8, 25, 26].

Glass ionomer cement (GI) specimens were prepared according to the manufacturer’s instructions. The powder and liquid were mixed using a plastic spatula at a ratio of 4.7 g:1 g, then packed into molds placed on a glass slab and covered with a Mylar strip. Another glass slab lined with a Mylar strip was placed on top of the mold. A standardized static load of 500 g was applied uniformly over the glass slab for the duration of the initial setting period to ensure intimate adaptation of the cement to the mold walls, eliminate internal voids and porosities, and extrude excess material laterally beyond the mold margins [27]. Following removal of the applied load, the extruded excess cement was carefully removed using a sharp scalpel blade held parallel to the specimen surface, ensuring that the trimming procedure did not engage or disturb the specimen surface itself. The mold was then carefully disassembled, and any residual flash at the specimen periphery was similarly trimmed with the scalpel blade. All glass ionomer cement specimens were immediately coated with a thin layer of surface varnish (G-Coat Plus; GC Corporation, Tokyo, Japan) to protect the freshly set material from early moisture uptake and desiccation during the initial setting phase. The specimens were allowed to set for 10 min at 37 °C, then removed from the molds.

The two resin composite restorative materials were prepared according to the manufacturers’ instructions. Each material was introduced into the Teflon mold in a single increment using a composite placement instrument and condensed gently to ensure complete adaptation to the mold walls and elimination of air entrapment. A Mylar strip was placed over the filled mold, followed by a glass slab, upon which a standardized static load of 500 g was applied prior to light-curing to extrude excess composite beyond the mold margins and ensure a flat, void-free specimen surface. While maintaining the load and Mylar strip in position, each specimen was light-cured for 20 s on the top surface. The load and glass slab were then carefully removed, the specimen was inverted, and a further 20 s of light-curing was applied to the bottom surface (40 s total per specimen), using a light-emitting diode (LED) curing unit (1000–1200 mW/cm²; Guilin Woodpecker Medical Instrument Co. Ltd., Guilin, China), in accordance with the manufacturers’ instructions. Following removal of the applied load, the excess composite extruded beyond the mold margins was removed by carefully sliding a sharp scalpel blade flush along the flat outer face of the assembled Teflon mold; residual peripheral flash following demolding was similarly trimmed. No additional finishing or polishing was performed following specimen preparation; the Mylar strip-pressed surface was retained as the standardized baseline surface for all experimental groups. 

The light intensity of the LED curing unit was verified using a spectroradiometer (Demetron Research Corp., USA) before commencement of specimen preparation, every 10 specimens during preparation, and at the end of the preparation session; all recorded values remained within the range of 1000–1200 mW/cm² throughout. Following light-curing, all resin composite specimens were stored in artificial saliva at 37 °C for 24 h prior to baseline measurements and commencement of immersion testing, in accordance with ISO 4049:2019 guidelines [28], to ensure complete post-polymerization, dimensional stabilization, and water sorption equilibration.

### Specimen grouping

A total of 135 specimens were prepared from the tested restorative materials and divided into three groups (*n* = 45) according to material type. Each group was further subdivided into three subgroups (*n* = 15) according to immersion medium: (1) artificial saliva only (control), (2) HerbaL Gummies dissolved in 1.5 mL artificial saliva, and (3) Centrum chewable tablets dissolved in 1.5 mL artificial saliva.

Artificial saliva was prepared using 2.38 g Na₂HPO₄, 0.19 g KH₂PO₄, and 8.00 g NaCl per liter of distilled water. The pH was adjusted to 6.75 ± 0.05 using phosphoric acid and verified with a calibrated pH meter (Jenway 3305, Bibby Scientific Limited, Stone, Staffordshire, UK), consistent with the mean resting salivary pH reported in the physiological literature and previously validated artificial saliva formulations used in dental materials research [29–31].

Each subgroup was further divided into three groups (*n* = 5) according to the testing method. Specimens were individually immersed and stored in an incubator (BTC Engineering, Cairo, Egypt) at 37 °C. Each specimen was immersed in 1.5 mL of solution in separate vials to simulate the salivary volume in children. Sample size justification is provided in Sect.  2.9.

### Immersion protocol

The pH of each multivitamin medium was determined at room temperature (23 ± 1 °C) immediately following dissolution of the respective multivitamin in 1.5 mL of artificial saliva utilizing a calibrated digital pH meter (Jenway 3305, Bibby Scientific Limited, Stone, Staffordshire, UK). Each preparation was measured in triplicate, and the mean value was recorded. The recorded pH values were 4.7 for HerbaL Gummies and 5.1 for Centrum chewable tablets. Specimens were immersed for 69 s (equivalent to 80 chewing cycles) in each vitamin medium and in artificial saliva [32]. Specimens were incubated at 37 °C to mimic intraoral temperature, and testing after 6 months allowed assessment of long-term surface changes.

The specimens were then rinsed with deionized water, and the immersion medium was replaced with artificial saliva for the remainder of the 24-hour period. This immersion cycle was designed to simulate the average daily consumption time of multivitamins by children. Control specimens were stored in artificial saliva throughout the study period, and the medium was refreshed daily. Surface hardness, surface roughness, and color change were evaluated after 6 months.

The immersion protocol was designed to simulate realistic oral exposure conditions. An immersion time of 69 s per day was calculated based on the chewing time for one gummy or tablet, derived from the findings of Fujiwara S. et al. [32], who reported that gummy candy requires approximately 80 chews before swallowing, with each chew lasting 0.87 s. Immersion volume of 1.5 mL artificial saliva was selected to correspond with the known salivary flow rate of 1.5 mL/min, and vitamins were renewed every 24 h to replicate daily multivitamin consumption [33–35].

### Surface hardness testing

Surface hardness was measured using a digital Vickers hardness tester (NEXUS 4000™; model no. 4503, INNOVATEST, Maastricht, Netherlands) with a load of 100 gf and a dwell time of 15 s [14]. Three indentations were made on each specimen surface, and the mean value was calculated. VHN values were automatically calculated from the indentation diagonal measurements using IMPRESSIONS™ software (Version 2.5.0, INNOVATEST Europe BV, Maastricht, Netherlands) supplied with the hardness testing system.

### Surface roughness testing

Quantitative evaluation of surface roughness was performed using an atomic force microscope (Anton Paar Tosca 200 AFM, Anton Paar USA Inc., Ashland, VA, USA) in tapping mode. A scanning area of 100 × 100 nm was selected to capture nanoscale surface topographic changes at a resolution commensurate with the filler particle dimensions of the tested nanofilled restorative materials, consistent with previously reported AFM methodologies for nanofilled dental composites [36, 37].

Scans were obtained at three different locations on each specimen surface to ensure representativeness of the surface topography measurements. The arithmetical mean height (Sa) was selected over the conventional two-dimensional Ra parameter, as Sa provides a more comprehensive and accurate areal assessment of surface topography at the nanoscale using AFM [38]. Qualitative three-dimensional surface topography images were generated and analyzed using the Tosca Analysis Software (Anton Paar USA Inc., Ashland, VA, USA), which was also used to calculate the average surface roughness (Sa) values.

### Color change measurement

Prior to each measurement session, all specimens were removed from their immersion vials, gently rinsed with deionized water for 30 s to remove any residual solution or loosely adherent debris, and carefully blotted dry with absorbent paper to remove surface moisture.

Color change measurement was performed using the CIE L*a*b* color system with a spectrophotometer (Agilent Cary 5000, Agilent Technologies, Santa Clara, CA, USA), selected for its sensitivity, objectivity, and reproducibility in dental materials research [39]. In this system, L* represents the lightness component (0 = black; 100 = white), a* represents the red–green chromaticity axis (positive values = red; negative values = green), and b* represents the yellow–blue chromaticity axis (positive values = yellow; negative values = blue).

All color measurements were performed against a standardized white background (L* = 93.2, a* = −1.1, b* = 2.9), verified prior to each measurement session using certified calibration tiles supplied by the instrument manufacturer. A white background was selected in accordance with ISO 7491:2000 guidelines and is widely adopted in dental color stability research as it minimizes the influence of ambient light scatter and provides a high-contrast, reproducible optical reference condition [25, 26].

L*, a*, and b* values were recorded for each specimen before immersion and after 6 months of immersion. Color change (ΔE) was calculated using the following equation: ΔE = [(ΔL*)² + (Δa*)² + (Δb*)²]½, where ΔL*, Δa*, and Δb* represent the differences between baseline and post-immersion L*, a*, and b* values, respectively [25].

### Statistical analysis

Statistical analysis was performed using IBM SPSS Statistics version 25 (IBM Corp., Armonk, NY, USA). Quantitative data were expressed as mean ± standard deviation (SD). Data normality was assessed using the Shapiro–Wilk and Kolmogorov–Smirnov tests, and homogeneity of variances was confirmed using Levene’s test. As all data met parametric assumptions, parametric tests were applied. For each outcome variable (color change [ΔE], surface hardness [VHN], and surface roughness [Sa]), a two-way analysis of variance (two-way ANOVA) was conducted to evaluate: (1) the main effect of restorative material type; (2) the main effect of immersion medium; and (3) the interaction effect between material and immersion medium. When significant main or interaction effects were detected, post hoc pairwise comparisons were performed using Tukey’s HSD test.

Sample size was calculated a priori using G*Power software (Version 3.1) for a two-way ANOVA fixed effects model. Based on previous in vitro studies of restorative materials under immersion conditions, a large effect size (f = 0.40) was assumed. With α = 0.05 and 80% statistical power, the minimum required total sample size was 36 specimens. To ensure balanced allocation across the 3 × 3 factorial design, five specimens were assigned per cell, yielding 45 specimens in total. This sample size provided adequate power to detect significant main and interaction effects. The calculated minimum sample size (*n* = 45) applied per restorative material group; therefore, across three materials, the total sample size was 135 specimens.

Effect size was calculated for each factor in the two-way ANOVA model using partial eta-squared (η²), interpreted according to conventional benchmarks: small (η² = 0.01), medium (η² = 0.06), and large (η² ≥ 0.14). To statistically examine the relationship between surface roughness and color change, a Pearson product-moment correlation analysis was performed between Sa and ΔE values across all material–medium group means. The strength of correlation was interpreted as weak (*r* < 0.30), moderate (0.30–0.59), strong (0.60–0.79), or very strong (*r* ≥ 0.80). Statistical significance was set at *p* ≤ 0.05 for all analyses.

**Fig. 1:**
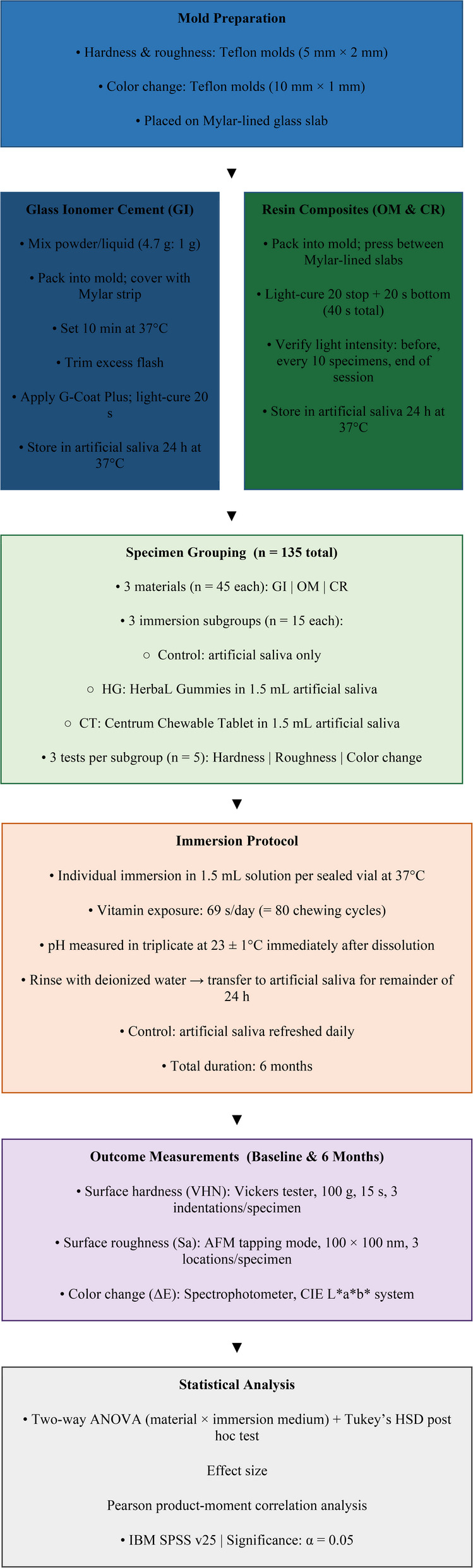
Flow diagram of specimen preparation and immersion protocol.Abbreviations: GI = glass ionomer cement; OM = Omnichroma; CR = Filtek Z350 XT composite resin; HG = HerbaL Gummies; CT = Centrum Chewable Tablet; VHN = Vickers hardness number; Sa = average surface roughness; ΔE = total color change; AFM = atomic force microscope

The complete experimental workflow, including specimen preparation, grouping, immersion protocol, outcome measurements, and statistical analysis, is summarized in Fig. 1.

## Results

### Surface hardness test results

Two-way ANOVA revealed significant main effects of restorative material, immersion medium, and a significant material × medium interaction for surface hardness (all *p* < 0.001), confirming that hardness reduction was material dependent. Full ANOVA parameters, effect size analysis and descriptive data are presented in Table 3.

Both multivitamin solutions significantly reduced surface hardness relative to control across all materials. Glass ionomer cement showed the greatest susceptibility, with HerbaL Gummies producing significantly lower VHN than Centrum (*p* < 0.001). Omnichroma responded comparably to both solutions (*p* = 0.524), whereas Filtek Z350 XT showed a greater reduction with HerbaL Gummies than Centrum (*p* = 0.014). Across all immersion conditions, Filtek Z350 XT maintained the highest VHN values, followed by Omnichroma and glass ionomer cement (all between-material comparisons *p* < 0.001).

**Table 3:** Two-way ANOVA results, effect size analysis and descriptive statistics (mean ± SD) for surface hardness (VHN)

Source / Variable	GI	Omnichroma	3 M Z350	*p* (between materials)	SS	df	F / Sig.	Effect Size (Partial η²)
Restorative Material				F = 215.14, *p* < 0.001*	10955.213	2	215.14 / <0.001*	0.68 (Large)
Immersion Medium				F = 99.34, *p* < 0.001*	5058.576	2	99.34 / <0.001*	0.31 (Large)
Material × Medium				F = 9.90, *p* < 0.001*	1008.355	4	9.90 / <0.001*	0.06 (Small-Medium)
Control	65.47 ± 4.87 aC	79.03 ± 4.27 aB	98.02 ± 4.78 aA	< 0.001*				
HerbaL Gummies	26.35 ± 5.43 cC	63.35 ± 3.07 bB	74.97 ± 6.62 cA	< 0.001*				
Centrum	53.68 ± 6.73 bC	65.79 ± 2.86 bB	86.99 ± 5.30 bA	< 0.001*				
*p* (within material)	< 0.001*	< 0.001*	< 0.001*					

Lowercase superscript letters within the same column indicate significant differences between immersion media within each material. Uppercase superscript letters within the same row indicate significant differences among materials within the same immersion condition

SS = Type III sum of squares,df = degrees of freedom

* Significant at *p* ≤ 0.05

### Surface roughness test results

Two-way ANOVA demonstrated significant main effects of restorative material, immersion medium, and a significant material × medium interaction for surface roughness (all *p* ≤ 0.001), indicating material-specific roughening responses. Full ANOVA parameters, effect size analysis and descriptive data are presented in Table 4.

**Fig. 2:**
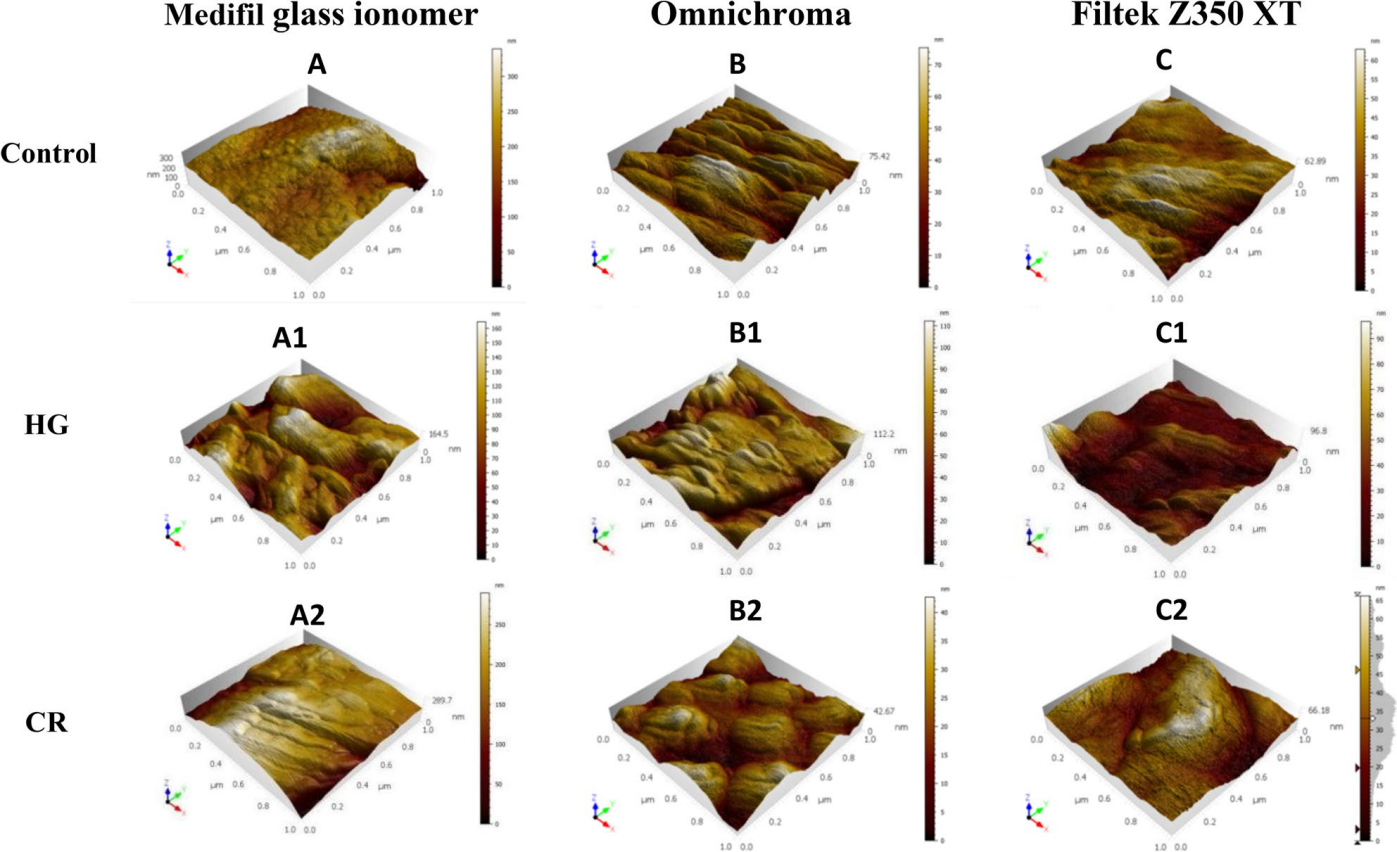
AFM 3D roughness profile of the tested materials after immersion in artificial saliva (control), HerbaL Gummies vitamin (HG) and Centrum chewing tablet (CT): (A- A2) for Medifil glass ionomer, (B-B2) for Omnichroma and (C-C2) for Filtek Z350 XT

Both solutions significantly increased surface roughness relative to control across all materials. For glass ionomer cement and Filtek Z350 XT, HerbaL Gummies and Centrum produced comparable roughening (*p* = 0.702 and *p* = 0.297, respectively), regardless of their pH difference. Omnichroma, however, exhibited significantly greater roughness with HerbaL Gummies than Centrum (*p* < 0.001), suggesting a material-specific threshold response. Glass ionomer cement consistently showed the highest Sa values under all conditions (*p* < 0.001 vs. both composites), while Omnichroma and Filtek Z350 XT differed significantly only under HerbaL Gummies immersion (*p* = 0.035). Three-dimensional AFM images corroborated these findings, with control specimens showing uniform surfaces and multivitamin-exposed specimens displaying progressive surface irregularity (Fig. 2).

**Table 4:** Two-way ANOVA results, effect size analysis and descriptive statistics (mean ± SD) for surface roughness (Sa, nm) among the tested restorative materials under different immersion conditions

Source / Variable	GI	Omnichroma	3 M Z350	*p* (between materials)	SS	df	MS	F / Sig.	Effect Size (Partial η²)
TWO-WAY ANOVA					ANOVA Parameters				
Restorative Material				F = 864.24, *p* < 0.001*	4394.926	2	2197.463	864.24 / <0.001*	0.91 (Large)
Immersion Medium				F = 84.35, *p* < 0.001*	428.958	2	214.479	84.35 / <0.001*	0.09 (Medium)
Material × Medium				F = 6.20, *p* = 0.001*	63.085	4	15.771	6.20 / 0.001*	0.01 (Small)
Error					91.536	36	2.543		
DESCRIPTIVE STATISTICS — Mean ± SD (Sa, nm)									
Control	24.76 ± 3.36 bA	6.64 ± 1.45 cB	7.08 ± 0.09 bB	< 0.001*					
Jelly Vitamin	34.66 ± 1.16 aA	14.25 ± 0.51 aB	11.57 ± 2.22 aC	< 0.001*					
Centrum	33.56 ± 0.99 aA	10.51 ± 1.15 bB	10.22 ± 0.80 aB	< 0.001*					
*p* (within material)	< 0.001*	< 0.001*	< 0.001*						

Lowercase superscript letters within the same column indicate significant differences between immersion media within each material. Uppercase superscript letters within the same row indicate significant differences among materials within the same immersion condition

SS = Type III sum of squares; df = degrees of freedom; MS = mean square

* Significant at *p* ≤ 0.05

### Color change results

Two-way ANOVA showed significant main effects of restorative material, immersion medium, and a significant material × medium interaction for color change (all *p* < 0.001), indicating that color response to immersion media was material dependent. Full ANOVA parameters, effect size analysis and descriptive data are presented in Table 5.

Both solutions significantly increased ΔE relative to control across all materials. For glass ionomer, HerbaL Gummies and Centrum produced equivalent color change (*p* = 0.999), whereas both composite materials exhibited significantly greater color alteration with HerbaL Gummies than Centrum (Omnichroma: *p* = 0.020; Filtek Z350 XT: *p* < 0.001). Omnichroma consistently showed the highest ΔE values across conditions, except under Centrum where it did not differ significantly from Filtek Z350 XT (*p* = 0.196). Notably, all recorded ΔE values remained below the clinically acceptable threshold of 3.3, suggesting that the changes, while statistically significant, are unlikely to be clinically perceptible.

Pearson correlation analysis performed between Sa (surface roughness) and ΔE (color change) values across all material–medium combinations (*n* = 9 group means), revealed a statistically significant strong positive correlation between surface roughness and color change (*r* = 0.81, *p* = 0.008), indicating that materials and conditions associated with greater surface roughening also exhibited greater color alteration. This finding provides quantitative statistical support for the mechanistic relationship between acid-induced surface degradation, microporosity development, and enhanced chromogen penetration.

**Table 5:** Two-way ANOVA results, effect size analysis and descriptive statistics (mean ± SD) for color change (ΔE)

Source / Variable	GI	Omnichroma	3 M Z350	*p* (between materials)	SS	df	F / Sig.	Effect Size (Partial η2)
Restorative Material				F = 415.29, *p* < 0.001*	6.587	2	415.29 / <0.001*	0.27 (Large)
Immersion Medium				F = 853.90, *p* < 0.001*	13.545	2	853.90 / <0.001*	0.55 (Large)
Material × Medium				F = 13.57, *p* < 0.001*	0.430	4	13.57 / <0.001*	0.02 (Small)
Control	1.03 ± 0.02 bC	1.69 ± 0.02 cA	1.39 ± 0.02 cB	< 0.001*				
HerbaL Gummies	1.96 ± 0.08 aC	3.13 ± 0.11 aA	2.75 ± 0.08 aB	< 0.001*				
Centrum	1.95 ± 0.13 aB	2.92 ± 0.15 bA	2.43 ± 0.07 bA	< 0.001*				
*p* (within material)	< 0.001*	< 0.001*	< 0.001*					

* Significant at *p* ≤ 0.05

## Discussion

The null hypothesis was rejected, as both tested multivitamin formulations significantly affected the surface hardness, surface roughness, and color stability of all evaluated restorative materials. These findings indicate that commonly consumed pediatric multivitamins may compromise the surface integrity and esthetic properties of tooth-colored restorations under simulated long-term exposure conditions, with the extent of degradation varying according to material composition and the specific multivitamin formulation used.

The 69-second daily immersion duration was derived from the study by Fujiwara et al. [32], who reported that consumption of one gummy candy requires approximately 80 chewing cycles, with each cycle averaging 0.87 s, yielding a total oral contact time of approximately 69 s per supplement dose. Over the 6-month study period, this equates to a cumulative acid exposure time of approximately 207 min (3.45 h), distributed as once-daily supplementation, a regimen consistent with standard pediatric multivitamin dosing guidelines. It is important to recognize that this represents a repeated, uninterrupted acid challenge at a pH well below the erosive threshold of 5.5, delivered directly to the restoration surface in a confined salivary volume of 1.5 mL and are often administered as a standalone dose, maximizing acid contact time with restorations. The significant surface degradation observed after 6 months of this protocol underscores the erosive relevance of even brief but repeated daily acid exposures on restorative materials in the pediatric oral environment. 

Regarding surface hardness, the greater reduction observed in Medifil glass ionomer cement following HerbaL Gummies immersion compared to Centrum can be mechanistically explained by the higher citric acid concentration and lower pH of the gummy formulation, which accelerates H⁺ ion diffusion into the glass ionomer cement matrix. This ionic exchange displaces divalent metal cations from the polyacrylate cross-links, progressively weakening the matrix and reducing its hardness. As confirmed by Gurdogan Guler EB et al. [1], prolonged exposure to acidic supplements produces a softening effect on glass ionomer cement surfaces, with the degree of softening proportional to the acidity and composition of the immersion medium. For Filtek Z350 XT composite resin, the greater hardness reduction induced by HerbaL Gummies relative to Centrum may be attributed to the hydrophilic nature of the TEGDMA monomer component. TEGDMA promotes water sorption, which facilitates plasticization of the polymer network and leaching of unreacted monomers, ultimately reducing the degree of cross-linking and hardness [40, 41]. The more aggressive acidic environment created by HerbaL Gummies likely accelerated this process compared to Centrum. In contrast, Omnichroma demonstrated comparable hardness values following immersion in both multivitamin solutions, which may be related to its unique filler system comprising uniformly sized 260 nm spherical SiO₂-ZrO₂ particles. The structural homogeneity and high filler density of these nanoparticles, combined with the UDMA-based resin matrix, may provide greater resistance to differential acid attack, thereby limiting the influence of pH variation between the two tested solutions. 

The greater susceptibility of glass ionomer cement to hardness reduction compared with composite resin materials is consistent with findings reported across multiple in vitro investigations. Gurdogan Guler EB et al. [1], evaluated the effect of pediatric multivitamin syrups and effervescent tablets on the surface microhardness and roughness of a conventional glass ionomer cement (Ketac Molar) and a giomer (Beautifil II), demonstrating significant hardness reductions in both materials following immersion in all tested multivitamin preparations over a 28-day period. Their findings confirm that acidic multivitamin preparations exert a measurable softening effect on glass ionomer cement surfaces regardless of formulation type. Similarly, Wongkhantee et al. [14], reported that exposure to acidic food and drinks significantly reduced the surface hardness of tooth-coloured filling materials including glass ionomer cement, with the degree of hardness reduction being proportional to the acidity of the immersion medium.

Regarding composite resin materials, Hengtrakool et al. [42], demonstrated that immersion in naturally acidic agents produced significant hardness reductions in both glass ionomer cement and resin composite specimens, attributing the composite resin degradation to hydrolytic breakdown of the polymer matrix and filler-matrix interface disruption under acidic conditions. These mechanistic findings support the greater hardness reduction observed in Filtek Z350 XT following HerbaL Gummies immersion in the present study, where the more acidic environment likely accelerated TEGDMA-mediated water sorption and plasticization of the polymer network. Additionally, a recent systematic review by Kaur et al. [43], covering studies from 1990 to December 2024 confirmed that composite resins and modified glass ionomer cements demonstrate a definite risk of surface degradation associated with chronic exposure to acidic pediatric liquid medications, with the degree of degradation being material- and formulation-dependent.

Regarding Omnichroma specifically, Atabek et al. [44], evaluated the effect of simulated gastric acid on the surface roughness and microhardness of monochromatic composites including Omnichroma, reporting significant hardness reductions following acidic immersion at both 7 and 14 days. The comparable hardness values of Omnichroma following immersion in both multivitamin solutions may be attributed to its homogeneous spherical SiO₂-ZrO₂ filler system providing relatively uniform resistance to acid attack, a finding that partially contrasts with Atabek et al. [44], where Omnichroma demonstrated lower top surface microhardness than other monochromatic composites following gastric acid exposure. This discrepancy may reflect differences in the nature, pH, and titratable acidity of the immersion solutions used across the two studies, further highlighting that pH alone does not fully predict the extent of material degradation. 

The equivalent roughening effect of both multivitamin solutions on Medifil glass ionomer cement and Filtek Z350 XT composite resin suggests that the surface degradation threshold for these materials was reached by both solutions irrespective of their pH differences. This implies that the type and concentration of acidic constituents, rather than pH alone, governs the extent of surface deterioration. Acidic solutions are known to induce softening of composite resin surfaces through leaching of resin components and subsequent displacement of filler particles, resulting in surface porosity and increased roughness [39]. For glass ionomer, the extraction of metal cations from the polyacrylate matrix by H⁺ ions exposes undissolved glass particles at the surface, producing a protruded and irregular surface topography. These findings agree with Münchow E et al. [39], who demonstrated that the degradation of resin composite surfaces is governed by both the pH and the specific chemical nature of the immersion solution, as well as by the intrinsic material properties. Conversely, the greater surface roughness induced by HerbaL Gummies in Omnichroma compared to Centrum may be explained by the differential solubility of its spherical SiO₂-ZrO₂ nanofillers under more acidic conditions. The lower pH of HerbaL Gummies may have exceeded a critical threshold required to disrupt the filler-matrix interface in Omnichroma, resulting in greater particle displacement and a more heterogeneous surface topography compared to the milder acidic environment created by Centrum.

Although one might expect a direct correlation between solution pH and the degree of surface degradation, this assumption was not uniformly supported by the present findings. Both multivitamin solutions produced comparable effects on certain material-property combinations despite their different pH values, indicating that solution composition, including the type of organic acids, their chelating potential, and the solubility of the dissolved ionic species, plays an equally important role in determining material degradation. This is supported by previous studies demonstrating that the bond type of the resin matrix, the degree of monomer conversion, and solvent sorption uptake collectively influence the degradation rate of composite resins [39]. These findings highlight that clinical recommendations regarding the safety of acidic supplements for patients with tooth-colored restorations should not be based solely on pH values but should also account for the full chemical composition of the formulation.

The finding that glass ionomer cement consistently exhibited the highest surface roughness values under all immersion conditions is supported by the broader literature on acid-induced surface degradation. Aktaş et al. [45], investigated the effect of various syrup-formed dietary supplements on the surface roughness and color stability of seven direct restorative materials used in pediatric dentistry, reporting that the highest roughness values were consistently recorded in the conventional glass ionomer cement group across all tested supplements and immersion time points. This pattern is in direct agreement with the present findings and reflects the inherent susceptibility of the polyacrylate matrix of glass ionomer cement to H⁺ ion-mediated dissolution and cation leaching, which exposes undissolved glass particles at the surface and produces an irregular surface topography. Importantly, Aktaş et al. [45], further confirmed that all experimental groups in their study exhibited surface roughness values exceeding the clinically relevant threshold of 0.2 μm associated with bacterial plaque retention as established by Bollen et al. [15], a finding with direct clinical implications consistent with those of the present investigation.

The equivalent roughening effect of both multivitamin solutions on glass ionomer cement and Filtek Z350 XT, despite their different pH values, is consistent with findings reported by Gurdogan Guler EB et al. [1], who noted that the erosive potential of a solution is not solely dependent on pH but is also governed by titratable acidity, the type of organic acid, and the mineral content of the preparation. Hasan et al. [46], similarly examined the effect of acidic and basic pediatric medications on the surface roughness of glass ionomer restorative cements, confirming significant roughness increases following acidic medication exposure and highlighting that chemical composition rather than pH alone determines the magnitude of surface deterioration. With respect to Omnichroma, Vejendla et al. [47], evaluated the color stability and surface roughness of Omnichroma following immersion in alcoholic beverages, reporting significant increases in surface roughness after immersion, with the degree of roughening varying according to the specific immersion medium.

Furthermore, Yildirim et al. [26], comparing monochromatic composites including Omnichroma after immersion in staining solutions reported that Omnichroma exhibited lower surface roughness than Filtek Z350 XT after thermocycling and immersion, suggesting a degree of surface stability attributable to its uniform spherical filler system. The greater roughness induced by HerbaL Gummies compared to Centrum in Omnichroma in the present study suggests that more acidic conditions may exceed a threshold required to disrupt the filler-matrix interface of this material, a relationship not observed in the less complex acidic environment created by Centrum.

Color measurement using the CIELAB color system with the ΔEab formula is a standardized technique that predicts color changes in an objective manner. While the CIEDE2000 formula (ΔE00) has been reported to provide better correlation with human visual perception due to corrections for lightness, chroma, and hue non-uniformities, the ΔEab formula was selected for the present study to enable direct comparison with most of the existing dental materials literature, which predominantly employs ΔEab for color stability assessment. Both formulas demonstrate good agreement for small color differences, such as those observed in the present study, where all ΔE values remained below 3.3.

The significant color changes observed across all materials following multivitamin immersion may be attributed to surface degradation-induced microporosity, which facilitates the penetration and retention of chromogenic compounds present in the acidic solutions. The more pronounced color alteration in Omnichroma and Filtek Z350 XT following HerbaL Gummies immersion compared to Centrum may reflect the greater degree of surface degradation and increased surface roughness, which provides more sites for pigment adsorption and entrapment. The relatively equivalent color change of Medifil glass ionomer cement following both multivitamin solutions is consistent with the equivalent surface roughness produced by both solutions in this material, suggesting a direct relationship between surface topography and color susceptibility. Importantly, all observed ΔE values remained below the clinically acceptable threshold of ΔElab ≤ 3.3 [48], indicating that despite the statistically significant color differences, the esthetic impact of daily multivitamin consumption on these restorations may be considered clinically acceptable over a 6-month period. Nevertheless, long-term cumulative exposure warrants further investigation.

The Pearson correlation analysis confirmed statistically the mechanistic relationship between surface roughening and color susceptibility. Acid-induced microporosity increases the available surface area for chromogen penetration and entrapment, and the strength of this correlation across three chemically distinct restorative materials suggests it is a generalizable phenomenon rather than material specific. Clinically, these findings underscore the importance of maintaining smooth restoration surfaces in patients consuming acidic supplements, as surface degradation simultaneously elevates plaque retention risk and esthetic deterioration. This relationship further explains why Omnichroma exhibited the greatest overall ΔE, mirroring its roughness behavior across immersion conditions, and why glass ionomer cement remained highly color-susceptible despite equivalent roughening under both multivitamin solutions [49]. Color difference was quantified using the CIELab ΔEab formula, with a perceptibility threshold of ΔEab > 1.0 and a clinical acceptability threshold of ΔEab ≤ 3.3, as established by Ruyter et al. [48], and widely adopted in dental materials research. All recorded ΔEab values in the present study remained below this threshold, indicating that the observed color changes, while statistically significant, are unlikely to be clinically perceptible under routine conditions. It is acknowledged that the CIEDE2000 formula (ΔE00), with its corresponding clinical acceptability threshold of ≤ 1.8, operates within a perceptually uniform color space and provides superior correlation with human visual perception; however, ΔEab and ΔE00 thresholds are not directly interchangeable and conclusions drawn using one metric cannot be extrapolated to the other [50, 51].

In the present study, both composite resin materials exhibited greater color alterations than glass ionomer cement following HerbaL Gummies immersion, a pattern consistent with the well-documented susceptibility of resin-based materials to pigment uptake due to their polymer matrix and degree of water sorption. The relatively equivalent color change of glass ionomer cement following both multivitamin solutions is further supported by the equivalent surface roughness produced by both solutions in this material, suggesting a direct relationship between surface topography and chromogen penetration capacity, as previously proposed by Bagheri et al. [49], who demonstrated that increased surface roughness provides additional sites for pigment adsorption and entrapment in both composite resins and glass ionomer cements. 

Regarding Omnichroma’s color stability, Yildirim et al. [26], reported that Omnichroma demonstrated the lowest color change values among all tested composites including Filtek Z350 XT across all immersion solutions and time intervals in their study, attributing this superiority to its structural color technology which relies on physical light interaction rather than pigment-based opacity. This contrasts with the present study, where Omnichroma consistently exhibited the highest ΔE values across all immersion conditions. This discrepancy may be explained by the fundamentally different nature of the immersion solutions: staining solutions such as tea and coffee used in prior studies interact primarily with the surface via chromogen adsorption, whereas the acidic multivitamin solutions evaluated in the present study induce structural surface degradation that may disrupt the optical properties of Omnichroma’s light-interacting spherical filler system more profoundly than conventional staining mechanisms.

The large effect size values (η²) observed for both material type and immersion medium indicate that these factors exert a substantial influence on the surface and esthetic properties of restorative materials. From a clinical perspective, this suggests that material selection and exposure to acidic pediatric supplements are not minor contributors but rather key determinants of restoration longevity and performance.This study has several notable strengths. The use of a full 3 × 3 factorial two-way ANOVA design with formal a priori sample size calculation enabled evaluation of both main effects and interaction effects, providing a more complete and statistically rigorous picture of material–medium relationships than previously reported. The immersion protocol was carefully designed to simulate realistic oral exposure, incorporating evidence-based parameters for chewing duration, salivary volume, and daily vitamin renewal. The use of AFM with the Sa parameter for surface roughness assessment provided nanoscale precision superior to conventional two-dimensional profilometry. Three clinically relevant restorative materials with distinct chemical compositions were evaluated simultaneously, enabling direct comparison of their susceptibility to multivitamin-induced degradation. The 6-month testing period allowed assessment of cumulative long-term effects. Furthermore, the use of a spectrophotometer with the standardized CIELAB system ensured objective and reproducible color evaluation.

However, the present study has several limitations that should be acknowledged. The in vitro design may not fully replicate the complex oral environment, including saliva buffering capacity, pellicle formation, masticatory forces, thermal cycling, and individual variations in oral hygiene. Only two multivitamin formulations and three restorative materials were evaluated, limiting the generalizability of the findings to other brands and material types. Chewing was simulated by immersion time alone without mechanical stress, which does not account for the abrasive effects of mastication. Additionally, the conventional ΔEab formula was used for color difference calculation; although CIEDE2000 (ΔE00) has been reported to correlate more closely with human visual perception, ΔEab was retained to allow direct comparison with previously published studies. Identical shades were not available across all tested materials due to manufacturer-specific shade systems. Although color changes were calculated relative to each specimen’s baseline measurement (ΔE), slight variations in initial chromatic values and translucency characteristics may have influenced color susceptibility. Furthermore, specimens were prepared using the Mylar strip technique without additional mechanical finishing or polishing, which may not fully replicate the surface characteristics of clinically placed and finished restorations. Future studies incorporating standardized polishing protocols, a broader range of materials and multivitamin formulations, along with ΔE00 color evaluation, are warranted to validate and extend these findings in clinical settings.

## Conclusions

Within the limitations of the present in vitro study, daily exposure to acidic pediatric multivitamin supplements, both in gummy and chewable tablet forms, adversely affected the surface hardness, roughness, and color stability of all tested tooth-colored restorative materials under simulated oral conditions, with material- and formulation-specific degradation patterns. Glass ionomer cement demonstrated the greatest susceptibility to surface hardness reduction and roughening across all immersion conditions, while HerbaL Gummies exerted greater erosive potential than Centrum chewable tablets for most material–outcome combinations. Omnichroma exhibited a distinct response pattern attributable to its homogeneous spherical SiO₂–ZrO₂ nanofiller system, demonstrating comparable hardness values under both multivitamin solutions but the greatest color change across all conditions. Notably, all recorded color changes remained within clinically acceptable thresholds, indicating that esthetic compromise may not represent an immediate clinical concern over a 6-month exposure period; however, the observed surface hardness and roughness changes carry longer-term implications for restoration longevity and plaque retention. Further in vivo research incorporating a broader range of materials and multivitamin formulations is warranted to validate and extend these findings in clinical settings.

## Clinical implications

The acidic composition of pediatric multivitamin supplements should be considered when selecting restorative materials for children who routinely consume such preparations, given the material-specific susceptibility to surface degradation demonstrated in the present study. Where clinically feasible, chewable tablet formulations may be preferred over gummy vitamins for children with existing tooth-colored restorations, as the latter demonstrated greater erosive potential across most material–outcome combinations. Parents should be advised to administer multivitamin supplements with meals and to rinse children’s mouths with water immediately after consumption to minimize acid contact time with restorations. Regular clinical monitoring of restorations in children routinely consuming acidic supplements is recommended to ensure timely detection of surface degradation and to guide appropriate restorative management decisions.

## Data Availability

The data presented in this study are available on request from the corresponding author.
